# Structural definition of babesial RAP-1 proteins identifies a novel protein superfamily across Apicomplexa

**DOI:** 10.1038/s41598-023-49532-0

**Published:** 2023-12-15

**Authors:** Isidro Hötzel, Carlos E. Suarez

**Affiliations:** 1 Department of Antibody Engineering, Genentech, South San Francisco, CA 94080 USA; 2 Department of Veterinary Microbiology and Pathology, College of Veterinary Medicine, Washington State University, Pullman, WA USA; 3 Animal Disease Research Unit, United States Department of Agriculture - Agricultural Research Service, Pullman, WA USA

**Keywords:** Computational biology and bioinformatics, Microbiology, Molecular biology, Diseases

## Abstract

Apicomplexan protozoa are intracellular parasites of medical and economic importance. These parasites contain specialized apical complex organelles, including rhoptries, that participate in the process of host cell invasion. Conserved antigens expressed in the rhoptries are rational vaccine targets, but whether conservation of protein structure is a functional requirement for invasion remains unknown. Novel protein structural modeling enables identification of structurally conserved protein families that are not evident by sequence analysis alone. Here we show by AlphaFold2 structural modeling that the rhoptry-associated protein 1 superfamily of the Piroplasmida hemoparasites *Babesia* and *Theileria* (pRAP-1) is structurally conserved, with the core conserved region being composed of a globin-like and a 4-helix bundle subdomain. Search for structurally related members of this protein family in other apicomplexan parasites revealed structural homologues of pRAP-1 in several species of *Plasmodium*, *Toxoplasma gondii* and other members of the Sarcocystidae family. Based on these structural findings, pRAP-1 is a conserved apical complex protein, but whether these proteins share functional features in different species remains unknown. Identification of widely conserved elements involved in infection in these parasites will enhance our knowledge of invasion mechanisms, and facilitate the design of methods for controlling diseases that affect humans and animals globally.

## Introduction

Apicomplexan parasites are responsible for high impact diseases, such as malaria, babesiosis, theileriosis, and toxoplasmosis^1,2^. Parasites belonging to this phylum are defined by the presence of an apicoplast, a non-photosynthetic chloroplast remnant, and the apical complex structure^1^. This cellular structure is a highly evolved cell-invasion apparatus which is composed of rhoptries, micronemes and dense granules, also known as spherical bodies in *Babesia* parasites^1,3^. The process of cell invasion by most apicomplexans is not fully elucidated but involves the sequential secretion of microneme, rhoptry and dense granule contents upon parasite-host cell attachment^3,4^. The pear shaped rhoptries are a pair of secretory organelles rich in lipid and proteins that contain at least two defined compartments, the neck, and the main body. The protein contents of the rhoptries have been better characterized in *Toxoplasma* and *Plasmodium*, and it includes well-conserved pan-apicomplexan proteins mostly expressed in the rhoptry neck, such as the RON proteins, while less pan-conserved rhoptry proteins are localized in the main body of the organelle^3,4^. However, much less is known about the exact content of rhoptry proteins in *Babesia* and *Theileria* parasites.

The process of invasion usually includes initial recognition of the surface of the target cells by surface proteins followed by re-orientation of the parasite, resulting in the direct interaction or close juxtaposition of the apical complex end of the parasite with the cell membrane of the target cell^1,3,4^. A tight junction that contains microneme proteins discharged by the parasite is then formed between the parasite and the cell. As the parasite progresses in its self-propelled penetration, mediated by an actin-myosin motor inside the target cell, it forms a parasitophorous vacuole (PV) that separates the parasite from the host cell cytoplasm for as long as the parasite is inside the targeted cells in *Plasmodium* and *Toxoplasma* parasites. However, the PV dissolves quickly in *Babesia* and *Theileria* parasites, leaving these organisms in direct contact with the cytoplasm of the host cells^4–6^. Studies performed in *Toxoplasma* parasites indicated that rhoptry proteins are involved in the formation of the PV, but in most cases their functional role remains uncharacterized^3^. A common feature of rhoptry proteins is the presence of a signal peptide, while some are membrane proteins exposed on the surface of the merozoites. Thus, these proteins are potential targets for vaccines aimed at generating neutralizing antibodies that block invasion. In addition, some rhoptry proteins are known to be immunogenic and may potentially generate protective antibody responses in their hosts^2,7^. Most of these features are shared by the Rhoptry Associated Protein 1 family (RAP-1) proteins of *Babesia* and *Theileria* organisms^7^. These RAP-1 proteins, which are distinct and unrelated to the *Plasmodium* RAP-1 proteins, are of particular interest for vaccine development because they are highly conserved, surface-exposed, highly immunogenic, and contain neutralization-sensitive epitopes^8–13^.

The RAP-1 proteins of *Babesia* and *Theileria* contain a RAP-1 domain minimally defined by the presence of a conserved arrangement of four cysteines and a conserved 14 amino acid motif^8,9^. *B. bovis*, contains an unusual gene locus structure containing two identical *rap-1* genes organized in a head-to-tail tandem arrangement encoding two identical ~ 60 kDa proteins^12^ located in the chromosome IV of the parasite. In addition, the chromosome IV of *B. bovis* also contains a single-copy gene located in a different, but relatively close, locus encoding an RAP-1 related antigen (RRA), which also features the conserved RAP-1 domain^8,12^. The RRA protein was also identified in other *Babesia* parasites^4,7^. However, while the *B. bovis* RAP-1 proteins are highly immunogenic, the RRA is subdominant and elicits weaker immune responses^8^. In contrast, the highly complex *B. bigemina rap-1* locus contains 4 repeated and dimorphic versions of tandemly arranged *rap-1a* genes separated by intergenic regions containing the more conserved *rap-1b* genes, and a single copy *rap-1c* gene located terminally in the locus^14^. In addition, and similar to *B. bovis*, *B. bigemina* also contains a gene encoding the RRA protein in a separate, but again, relatively close locus (https://www.sanger.ac.uk/resources/downloads/protozoa/babesia-bigemina.html). This pattern of gene organization is similar in other known sensu stricto* Babesia* species^7^. However, *Theileria* parasites contain two or three head-to-tail tandemly arranged *rap-1* genes, encoding proteins with two or three RAP-1 domains each^15^. Remarkably, sensu lato* B. microti*, and most fully sequenced *Theileria* parasites, lack *rra* genes, which indicates *rra* as a possible marker for discriminating true *Babesia* parasites from sensu lato* Babesia* and *Theileria* organisms^7,15^.

Based on sequence analysis, it had been postulated that the *Babesia* and *Theileria* RAP-1 proteins were confined to the members of piroplasmids, such as *Theileria*, *Cytauxon* and *Babesia* parasites, and were thus renamed as pRAP-1 to differentiate them from the unrelated *Plasmodium* RAP-1 proteins^7^. Although not evident by amino acid sequence searches, the question remained of whether structural and/or functional homologues of the pRAP-1 proteins are also present and expressed in other important apicomplexan parasites, such as *Toxoplasma* and *Plasmodium* species.

Important insights about the function and conservation of proteins can be inferred from their structures, but experimental structural analysis can be cumbersome and costly and traditional in silico structural modeling methods inaccurate. The recent emergence and public availability of AlphaFold2 (AF2), a protein structure prediction system based on artificial intelligence, has greatly facilitated accurate protein structure predictions^16,17^.

In this study we performed structural modeling of babesial RAP-1 and RRA using AF2 to identify a conserved core RAP-1 domain with a globin-like subdomain linked to a helical bundle subdomain present in all Babesia RAP-1 and RRA molecules and the RAP-1 molecules expressed by *Theileria* species. The structural models reveal unexpected structural parallels between highly conserved RAP-1 regions and globin domain proteins. Using the RAP-1/RRA conserved structural domain to search the AlphaFold database of predicted protein structures resulted in the identification of RAP-1 structural homologues in both *Plasmodium* and *Toxoplasma* parasites. Since the members of the RAP-1 family have been regarded as important components of the invasion apparatus in *Babesia* and *Theileria* parasites^10,12,13,15^, they may also play similar functional roles in other important pathogens of humans and animals. Structural conservation of this superfamily of proteins among distinct apicomplexan parasites indicates that proteins containing the conserved RAP-1 domains may play important functional roles that are common to the phylum. The discoveries described here, including the presence of globin-like domains in apicomplexan parasites for the first time, may guide the design of experiments aimed at elucidating the functional role of RAP-1 like proteins in widely divergent apicomplexan parasites that can be the targets of novel therapeutic approaches against important diseases of humans and animals.

## Results

### Structural models of piroplasmid pRAP-1

The RAP-1 protein family of *Babesia* and *Theileria* species were modeled with AF2 using a publicly available server^18^. Protein sequences were used as input including the amino terminal secretion signal sequence. That region, usually modeled as a helix, was removed from the models for analysis. Similar models were also released in the publicly available AlphaFold database during this investigation^19^. However, the models obtained and described here are of generally better quality, as indicated by formation of disulfide bonds that are sometimes not observed in the public models.

**Figure 1:**
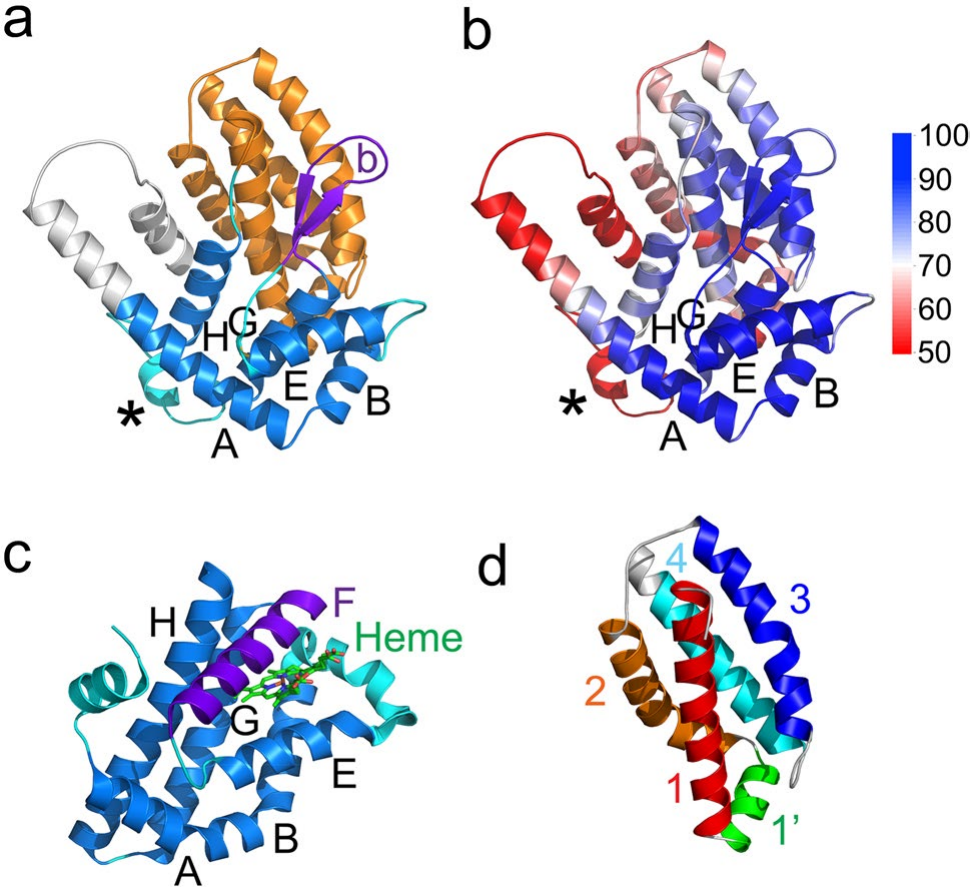
Structural model of the *B. bovis* RAP-1 domain. (**a**) Cartoon representation of the *B. bovis* RAP-1 model. Helical regions within the globin-like domain are shown in blue and labeled A to G. The hairpin loop within the globin-like domain is shown in purple and labeled with a “b”. The highly diverse RAP-1 region is shown in cyan and indicated by an asterisk. The 4-helix bundle region is shown in orange. The amino-terminal region with low quality-scores is shown in white. (**b**) pLDDT scores mapped on the *B. bovis* RAP-1 domain structural model. The model is shown and labeled as in (**a**), with the scores shown as indicated by the scale bar. (**c**) Globin domain of the globin-coupled sensor of *Geobacter sulfurreducens* (PDB accession 2W31), shown in a similar orientation as the model in panel (**a**), aligned through helices E. Helix F, which in *B. bovis* is replaced by the hairpin loop, is shown in purple. The helices with corresponding helices in *B. bovis* RAP-1 are shown in blue, with the region between helices B and E and an amino-terminal helix shown in cyan. The heme group is shown as sticks in green. (**d**) 4-helix bundle subdomain of the RAP-1 model. Sections of the helices that are structurally conserved between pirosplamida, *Plasmodium* and *Toxoplasma* RAP-1 are shown with different colors and labeled.

All sequences (see methods) yielded high-quality models of the RAP-1 domain with pLDDT scores often above 70 and 90, which indicate main-chain and side-chain reliability respectively^16^. The RAP1 domain is preceded and followed by low-quality modeled regions (pLDDT < 50) (Fig. 1b, Supplementary Fig. 1). The carboxy-terminal low-quality regions in the *B. bovis* RAP-1 model correspond to the sequences not conserved between species or orthologs within species^8^. The amino-terminal residues of the RAP-1 domain have low quality scores (pLDDT < 50) in models, with the first position with a pLDDT score greater than 70 in the *B. bovis* RAP-1 model being residue 52. As expected, the models of *Theileria* RAP-1 have 2 or 3 RAP-1 domains in tandem that seem to fold independently of each other (not shown).

**Figure 2:**
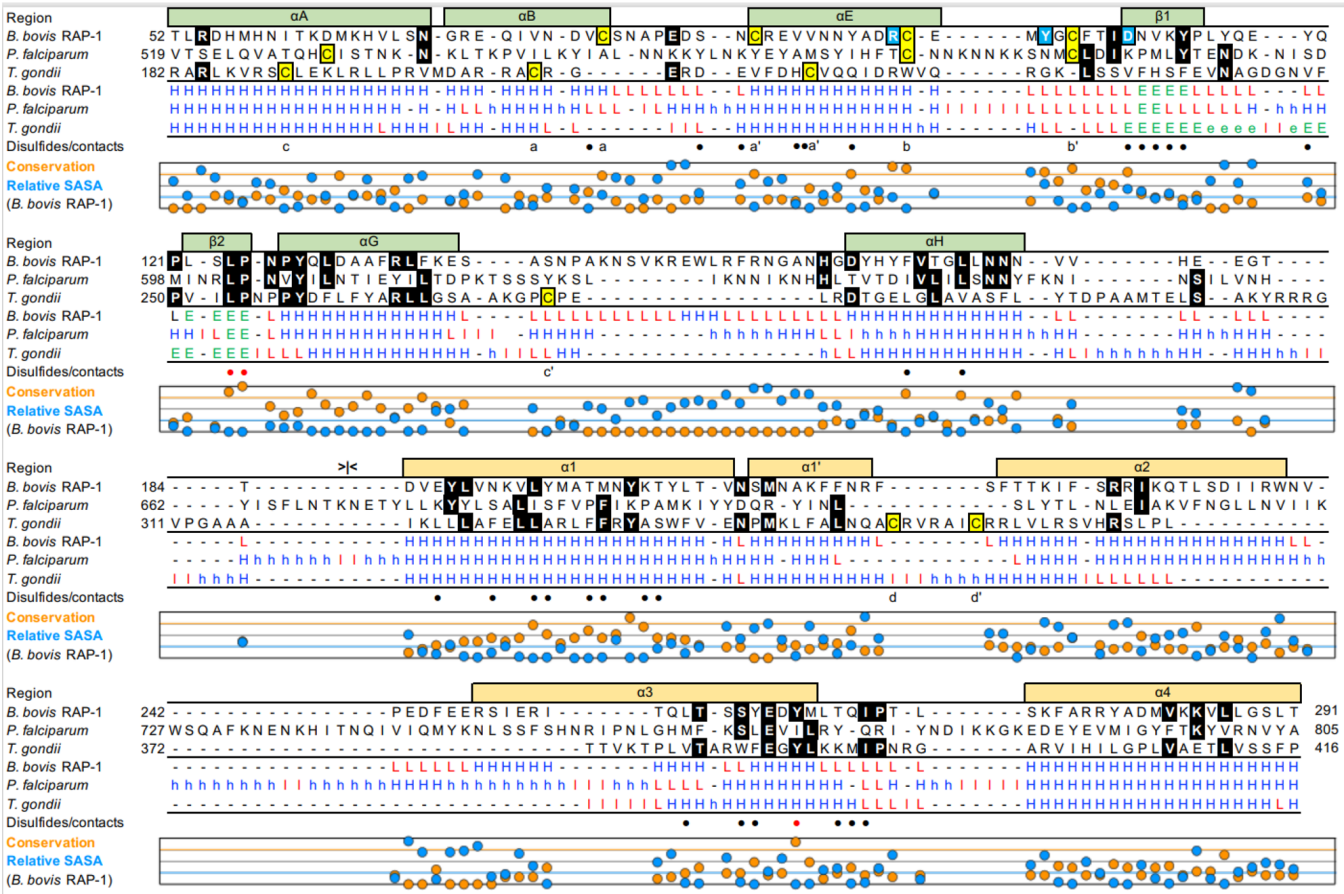
Structural alignment of the RAP-1 domain of *B. bovis*, *P. falciparum* and *T. gondii* pRAP1. Sequences were aligned structurally using the DALI server. Rows 1–3 show amino acid sequences and rows 4–6 show secondary structure of each residue, with helices, loops and extended regions shown as H (blue), L (green) and E (red). Structural elements shown in upper case are structurally aligned whereas lower-case indicates lack of alignment. Helices and beta-strands are shown above the alignments in green, within the globin-like domain, and yellow within the 4-helix bundle, with the approximate boundary between the two subdomains indicated with “ >|< ”. Disulfides are shown in row 7 below the alignments. Conservation of residues among Babesia and *Theileria* RAP-1 orthologs and relative solvent-accessible surface area (SASA) of each residue are shown in orange and blue, respectively, below the alignments in a scale of 0% to 100% for both. The orange and blue lines indicate 75% residue conservation and 25% SASA. Black and red dots in row 7 indicate residues involved in interactions between the globin-like and 4-helix bundle subdomains, with the red dots highlighting the 3 highly conserved Leu-124, Pro-125 and Tyr-263 residues. Conserved residues are highlighted in black and cysteines in yellow backgrounds. The 3 highly conserved Arg-99, Asp-109 and Tyr-103 surface residues are shown in light blue backgrounds.

The amino-terminal Babesial RAP-1 domain includes a total of 12 ɑ-helices (Figs. 1a and 2). These helices form two subdomains. The first subdomain adopts a fold similar to globin domains (Fig. 1a). One of the closest globin domain structural homologs of the RAP-1 globin-like domain in the Protein Data Bank is the globin domain of the globin-coupled sensor of *Geobacter sulfurreducens* (PDB accession 2W31, Z-score 3.6, root mean square deviation, 4 Å) (Fig. 1c). Following the nomenclature of globin domain helices, the RAP-1 globin-like domain has helices A, B, E, G and H (Figs. 1a and 2). All the *Babesia* and *Theileria* RAP-1 and RRA models are very similar (Supplementary Fig. 2). Therefore, we describe below the *B. bovis* RAP-1 model in detail following the residue numbering in this protein, highlighting the more significant differences in models of other members of this protein family.

A unique distinguishing feature of the RAP-1 globin subdomain is the replacement of helix F, which in globins usually coordinates a heme prosthetic group with helix B, with two antiparallel β-strands forming a hairpin loop conserved in all RAP-1 models (Fig. 1a,c). The region between helices G and H, which in some orthologs include one or more short helices, is the least conserved structurally in the globin-like subdomain and also has the lowest quality scores in the models (Fig. 1a,b), suggesting a variable and possibly flexible region within the globin-like subdomain. The four conserved RAP-1 cysteines form two disulfide pairs in a sequential configuration a/a’ and b/b’ (Fig. 2). These disulfides define the turns between helices B and E and after helix E. Helix A has variable length in different RAP-1 ortholog models, with the amino-terminal extension usually having a lower pLDDT score (Fig. 1b).

The second subdomain of the RAP-1 domain is a 4-helix bundle with an extra helix, ɑ-helix 1’, at the bottom of the domain (Fig. 1d). The 4 helices in the helix bundle are in a down-up-down-up configuration, with helices 1 and 2 in a parallel configuration with helices 3 and 4 respectively. In some models, helices 3 and 4 do not pack together with helices 1 and 2 and have lower quality scores. The third RAP-1 domain repeat of *T. equi* RAP-1 lacks the last two helices of the 4-helix bundle subdomain (not shown). The two subdomains are closely associated through hydrophobic interactions between ɑ-helices G and H and the hairpin loop in the globin-like domain and ɑ-helices 1 and 3 in the 4-helix bundle subdomain.

**Figure 3:**
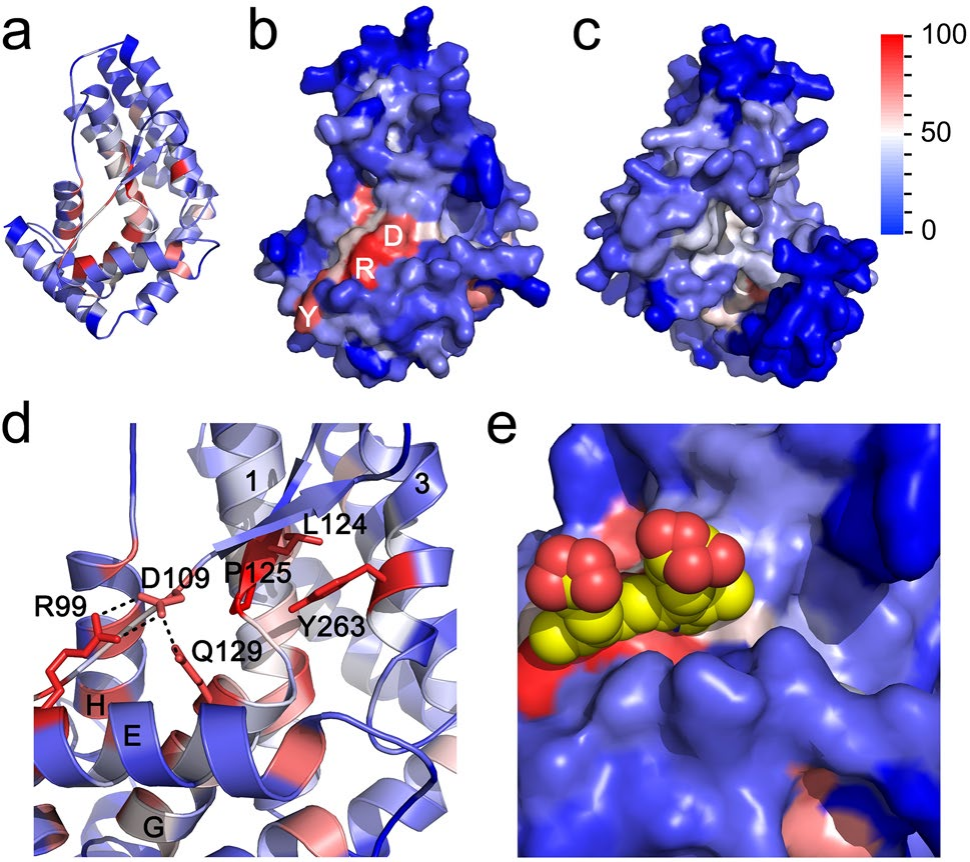
Sequence conservation of RAP-1. (**a**–**c**) Residue conservation, as shown in Fig. 2, mapped onto the *B. bovis* RAP-1 domain model, in cartoon (**a**) and surface (**b** and **c**) representation. The conservation of residues among *Babesia* and *Theileria* RAP-1 orthologues is shown by the scale on the right of panel (**c**). Panels a and b are shown in the same orientation, with panel (**c**) showing the opposite side of the model, with the low-quality score amino-terminal region omitted from the models. The position of highly conserved surface residues Arg-99, Asp-109 and Tyr-103 are indicated in (**b**). (**d**) Interactions between highly conserved residues in the *B. bovis* RAP-1 model. Residue conservation is shown on the same scale as for other panels, with the highly conserved Arg-99, Asp-109 and Gln-129 forming a hydrogen-bond network and Leu-124, Pro-125 and Tyr-263 shown as sticks. Helices E, G, H, 1 and 3 are indicated. (**e**) Detail of the correspondence of the heme of *G. sulfurreducens* domain sensor (PDB accession 2W31), shown as a space-filling model, and the highly conserved surface patch formed by residues Arg-99, Asp-109. The RAP-1 and *G. sulfurreducens* globin domain sensor is aligned through helices E.

The most conserved residues in the RAP-1 domain are located in the core of the protein (Fig. 3a). The surface of the RAP-1 domain, excluding the lower-confidence amino-terminal region of the model, includes residues that are poorly conserved between RAP-1 orthologs except for three highly conserved residues, Arg-99, Tyr-103 and Asp-109 (Fig. 3b,c). Interestingly, the region of highest sequence conservation corresponds to a previously described 14-mer sequence^9,20^ straddling β-strand 2 and ɑ-helix G almost completely buried in the protein core. Three of the most conserved residues of RAP-1, Leu-124 and Pro-125 in the 14-mer sequence and Tyr-263 in the 4-helix bundle, pack against each other in the core of the protein (Fig. 3d). Other previously described short, conserved motifs of RAP-1^20^ are also buried in the protein and pack against the 14-mer region in the core (not shown).

Two of three highly conserved residues that are exposed on the protein surface, Arg-99 and Asp-109, along with Gln-129 in the 14-mer region in ɑ-helix G form a hydrogen bond network (Fig. 3d). These 3 residues have pLDDT scores greater than 90 indicating high side-chain conformation reliability, supporting the presence of the hydrogen bond network in RAP-1. These 3 residues are strictly conserved in all *Babesia* and *Theileria* RAP-1 family members with the exception of Asp-109 in *B. divergens* RRA, which has Asn in that position (a conservative replacement).

The globin-like domain of RAP-1 does not seem to coordinate a prosthetic group as in other globin domains. Structural alignment of the *B. bovis* RAP-1 globin-like domain with the globin domain of the *G. sulfurreducens* globin-coupled sensor, indicates that the region in the RAP-1 model where a prosthetic group would reside does not form a cavity that would accommodate a ligand (Fig. 3e). Instead, the RAP-1 region corresponding to the location of Fe^2+^ at the center of the heme group is occupied by the conserved hydrogen bond network formed by residues Arg-99, Asp-103 and Gln-129 (Fig. 3e).

**Figure 4:**
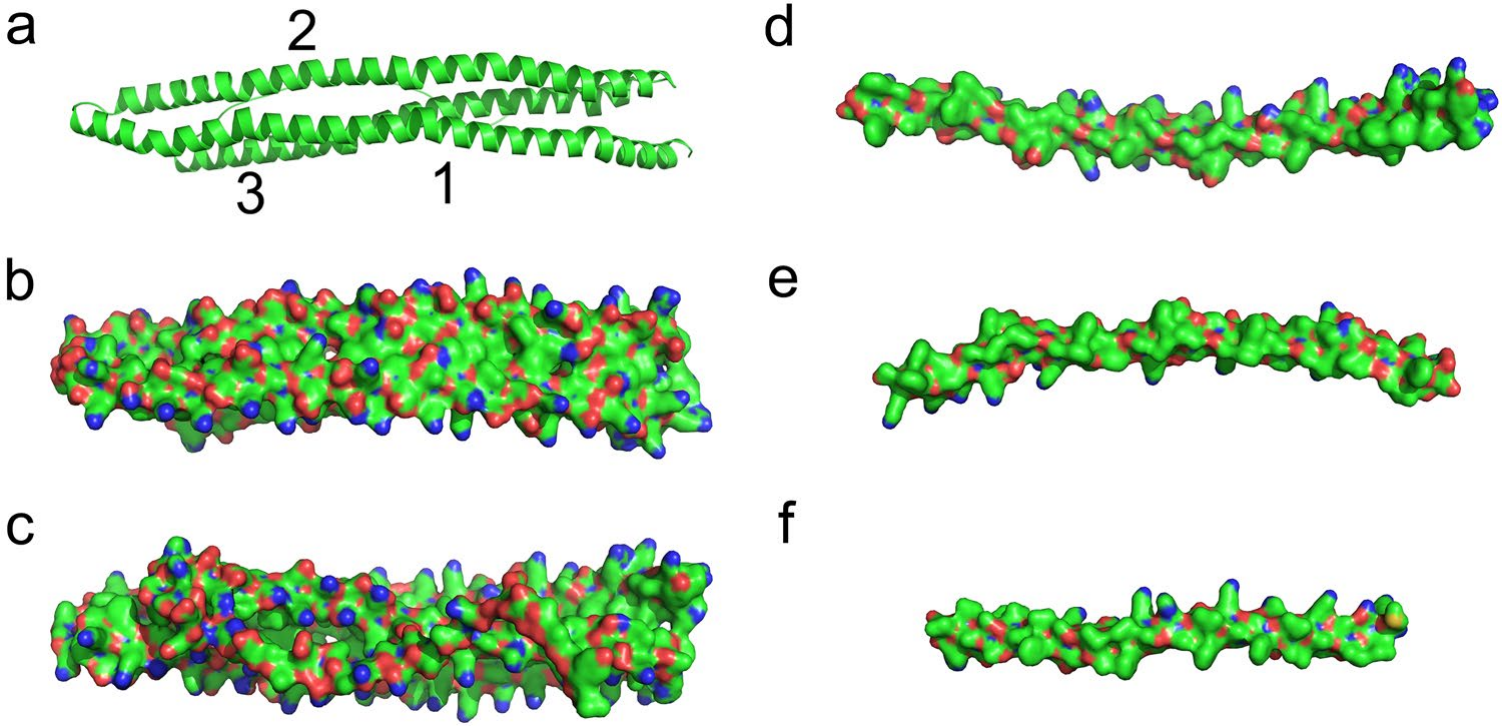
Carboxy-terminal *B. bovis* RAP-1 model. (**a**) Cartoon representation of the 3 helices of the repeat carboxy-terminal region outside the RAP-1 domain. (**b**) Same as (**a**), shown in a surface representation, with positively and negatively charged atoms shown in blue and red, respectively. (**c**) Opposite side of the model shown in panel (**b**), rotate around the x-axis. (**d**–**f**) Distribution of charges in the regions of helices 1, 2 and 3 of the carboxy-terminal regions facing the axis of the helix bundle, showing charges limited to main-chain atoms.

The carboxy-terminal regions of variable length that follow the conserved RAP-1 domain, which in some species like *B. bovis* includes several proline-rich repeats, have generally low-quality scores in the models and do not generally pack against the RAP-1 domain. In *B. bovis* RAP-1 the carboxy-terminal region forms three relatively long helices that pack against each other in a long 3-helix bundle (Fig. 4a). The three helices are amphipathic, with positions rich in hydrophilic and charged residues mostly in the exterior of the helix bundle and residues with hydrophobic side-chains mostly in the interior of the bundle (Fig. 4b–f). In several of the RAP-1 models in other species this region includes a variable number of predicted helical regions with a strong amphipathic configuration. While the global quality-scores of these helical regions are low in the models, the helical regions modeled in isolation have generally reliable quality scores (not shown).

### Identification of RAP-1 orthologs in Plasmodium

Orthologs of piroplasmid RAP-1 (referred here thereafter as pRAP-1, to differentiate from the unrelated plasmodial RAP-1 protein)^7^ have not been identified in *Plasmodium* based on sequence or other genomic-based searches. We used the pRAP-1 structural models to search for structural homologues in the AlphaFold database^17^ using the DALI server^21^. We used the globin-like domain of *B. bovis* RAP-1 without the 4-helix bundle for the searches to avoid hits with the relatively generic helical bundle structure and prioritize hits to the more specific and informative globin-like domain structure.

**Figure 5:**
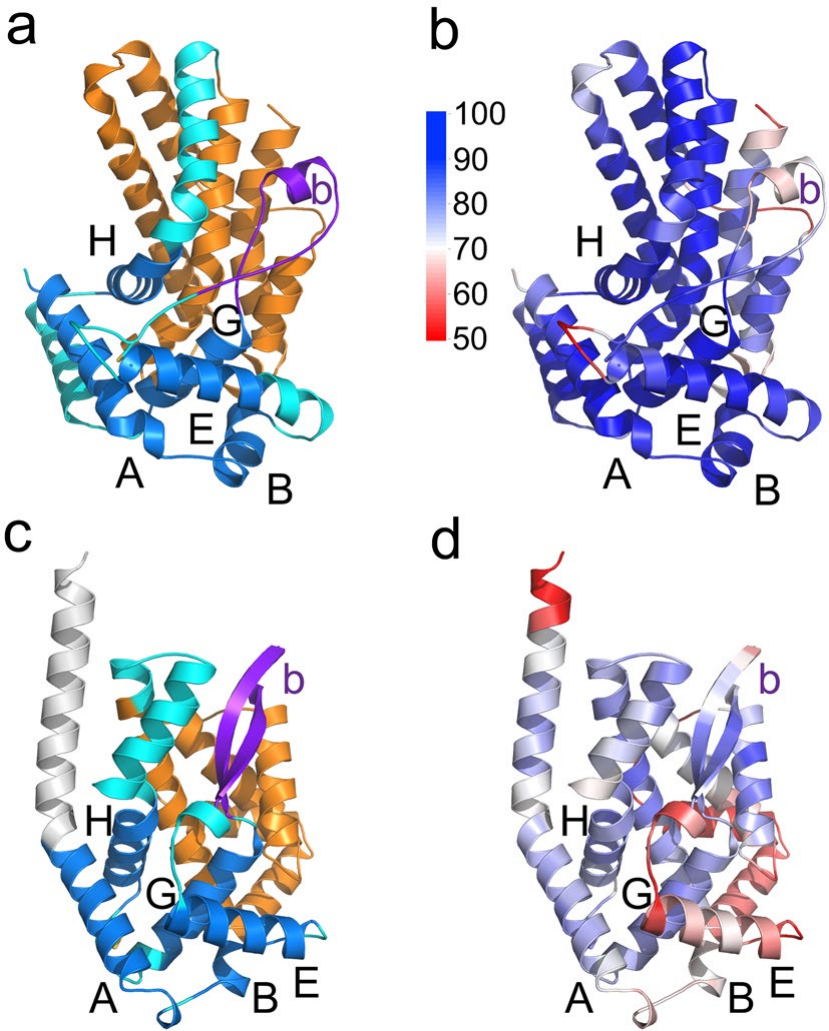
Structural models of *P. falciparum* and *T. gondii* pRAP-1 domains. (**a**) Cartoon representation of the *P. falciparum* Q8I4Y6 core domain model. The model is shown in the same orientation as the *B. bovis* RAP-1 domain in Fig. 1a. Helical regions within the globin-like domain are shown in blue and labeled A to G. The hairpin loop within the globin-like domain is shown in purple and labeled with a “b”. Regions that do not correspond to helices A-H are shown in cyan. The 4-helix bundle region is shown in orange. (**b**) pLDDT scores mapped on the *P. falciparum* Q8I4Y6 core domain model. The model is shown and labeled as in (**a**), with the scores shown as indicated by the scale bar. (**c**) and (**d**), same as (**a**) and (**b**), showing the model for *T. gondii* RAP-1 orthologue A0A151H8C6. The amino-terminal extension of helix A is shown in white.

A significant hit was identified in a high-quality model for Uniprot accession number Q8I4Y6 from *P. falciparum* isolate 3D7 (PF3D7_1244500) (Fig. 5a,b, Supplementary Fig. 2), encoded by gene PF3D7_1244500 (plasmodb.org). This protein, of unknown function, has a predicted amino-terminal secretion signal sequence. A homologue of this protein, also denominated PIMMS57, was recently found to be highly expressed in ookinete stages of *P. berghei* and likely involved in the ookinete to oocyst transition. Antibodies to the *P. berghei* homologue affect ookinete maturation and transmission^22^ and abolishment of transmission of gene KO parasite by mosquitoes^23^. It remains unknown whether this protein is associated with apical complex structures of the parasite or the possible role of the *P. falciparum* homologue. However, data in the *P. berghei* model suggests that this protein may be involved in interaction with the midgut during the process of midgut invasion by the parasite. Interestingly, it was also reported in this study that this protein contains Protein-Phosphatase 1c (PP1) binding domains^23^.

Although no sequence similarity can be detected between the *P. falciparum* Q8I4Y6 protein and any of piroplasmid pRAP-1 using BLAST, its pRAP-1 domain has all the structural features of the unique globin-like domain of pRAP-1 as well as a slightly modified 4-helix bundle following the globin-like domain. Structural alignment of the RAP-1 domain of this model with *Babesia* and *Theileria* pRAP-1 yielded highly significant alignments with Z-scores between 9 and 12.4 (Supplementary Fig. 2). Structural similarities included two extended anti-parallel regions in the place of helix F of globin domains and a degenerate 14-mer region in the same location as in pRAP-1. Given these very specific similarities between Q8I4Y6 and pRAP-1, Q8I4Y6 represents a structural orthologue of pRAP-1 in *Plasmodium*. Three significant differences are noted in Q8I4Y6 relative to other pRAP-1 family members. First, only the second pair of cysteines is present in the globin-like domain of Q8I4Y6. In addition, instead of the carboxy terminal extension that is observed in *Babesia* and *Theileria,* Q8I4Y6 has an amino-terminal extension of about 500 residues of low sequence complexity and a disordered structure in the models (Supplementary Fig. 1c). Finally, the helical bundle region of Q8I4Y6 has a slightly modified topology compared piroplasmid pRAP-1 with an extra well-defined helix before helix 1 and a split helix 3 that wraps around helix 4 with a connecting loop.

Additional conserved sequence elements in Q8I4Y6 include an isoleucine residue in the third helix of the bundle that packs against the 14-mer homologue in the same manner as Tyr-263 in piroplasmid pRAP-1. Residues corresponding to piroplasmid pRAP-1 Arg-99, Asp-109 and Gln-129 do not form a hydrogen bond network. Instead, in the same region of the surface, residues, Tyr-560, Tyr-564 and Ile-607 pack together through hydrophobic interactions. Of these three residues, only Ile-607 is a structural equivalent of Gln-129, while the tyrosine residues, while also present within ɑ-helix E, do not correspond to piroplasmid RAP-1 residues 99 and 109. Sequence homology searches using BLAST revealed that the *P. falciparum* pRAP-1 structural homologue is highly conserved in the genus *Plasmodium* as well as in related *Hepatocystis* species.

### Identification of members of the pRAP-1 family in *Toxoplasma gondii* and other members of the Sarcocystidae

We addressed whether pRAP-1 is also conserved in other members of the Apicomplexa that do not infect blood cells or require an arthropod definitive host. Searches of the AlphaFold database^24^ using the globin-like domain of Q8I4Y6 with the FoldSeek server^24–26^ identified a high-quality model of an uncharacterized putative transmembrane protein of *T. gondii* isolate TgCatPRC2 (Uniprot accession number A0A151H8C6) (Fig. 5c,d, Supplementary Fig. 2d). Homologues in *Neospora caninum* (Uniprot accession F0VNB0) and *Cystoisospora suis* (Uniprot accession A0A2C6KFA2, also a predicted secreted protein with a signal peptide), were identified by sequence homology searches. Models of the pRAP-1 homologues in the three species include all the features of pRAP-1 including the globin-like and 4-helix bundle subdomains. As in the piroplasmids and *Plasmodium*, the pRAP-1 structural orthologues of *Toxoplasma*, *Neospora* and *Cystoisospora* have a hairpin loop in place of the globin domain loop F. The conserved 14-mer homologue is also observed in these species, with the most conserved region being the PNP sub-motif. In fact, the *T. gondii* and *P. falciparum* model structures extend the conserved motif by three residues to encompass almost the entire ɑ-helix G (Fig. 2). Structural alignments between the RAP-1 domains of *Toxoplasma*, *Neospora* and *Cystoisospora* pRAP-1 and the pRAP-1 of *Plasmodium* and piroplasmids had significant Z-scores between 5.1 and 10.4 (Suppl. Figure 2).

A pair of cysteines forms a disulfide bond between ɑ-helices B and E although these cysteines are not structurally equivalent to piroplasmid pRAP-1 cysteines forming a disulfide bond between these two ɑ-helices. Two other disulfide bonds, one within the globin-like domain and another in the 4-helix bundle, are present in the *Toxoplasma*, *Neospora* and *Cystoisospora* pRAP-1 homologues. The topology of the helical bundle domain is slightly modified from both piroplasmid and *Plasmodium* pRAP-1 structural homologue, with a shorter helix 2 that has a different position in the bundle relative to other species. The residues corresponding to *B. bovis* RAP-1 residues 99, 109 and 129 do not form a hydrogen bond network, with some of the residues corresponding to that triad forming hydrogen bonds with other residues but not within a single network. The helix bundle within the RAP-1 domain in these 3 species is followed by a relatively short, unfolded extension with a helical region with amphipathic side-chain distribution.

## Discussion

Strong conservation of members of the RAP-1 superfamily among *Babesia*, *Theileria* and other piroplasmida parasites, strongly suggests functional relevance. Due to their localization within rhoptries, it is likely that RAP-1 functions are related to invasion of target cells by these parasites, which is also supported by experimental evidence using invasion neutralization assays^8,10^. However, specific RAP-1 functions remain unknown, and the discovery of the possible functions associated to these molecules can open new avenues to our understanding of the biology of these parasites and to new options for controlling important diseases affecting humans and animals, such as babesiosis, toxoplasmosis, malaria and theileriosis. Importantly, the definition of the three-dimensional structure of proteins provides critical insights into its function and a more sensitive way to identify possible functional homologues than approaches based solely on sequence searches. While experimental structure determinations remain cumbersome, structural prediction methods like AF2 are revolutionizing the field, facilitating accurate structure prediction and comparisons of many proteins. The use of this approach in this study yielded interesting findings and observations that can shed light on the function and extent of the members of the piroplasmid RAP-1 superfamily.

Structurally, the most surprising finding derived from this study was the presence of a globin-like domain in pRAP-1. This is the first instance of a globin-like domain, a relatively common fold in metazoans and bacteria, in Apicomplexan parasites. However, the globin-like domain of RAP-1 has a unique characteristic that distinguishes it from other globin-like domains, the replacement of ɑ-helix F by a β-hairpin loop, perhaps a key feature related to functional requirements. In globins, ɑ-helices F and E coordinate binding of a ligand. None of the characteristic residues coordinating heme prosthetic groups that are found in globins are evident in pRAP-1 family members. In addition, the region where the prosthetic group would be located does not form a cavity that would be compatible with ligand binding. Therefore, it is unlikely that pRAP-1 coordinates a heme group or another prosthetic group. Instead, in piroplasmid RAP-1 the space corresponding to the active center of a heme group is occupied by a hydrogen bond network that involves 3 of the most conserved RAP-1 residues, including the 2 most conserved surface residues of RAP-1. The structural conservation of that network on an exposed region that is functionally critical in globins supports the triad as a functionally important region on the RAP-1 surface. The exact function of that triad remains unclear, but, in analogy with other globin proteins, it could have sensor functions. Sensor functions have been proposed for other globins that do not coordinate ligands, including potential protein–protein interactions^26^. The function of the triad, as well as the role of each amino acid involved, could be analyzed in the future by designing stable transfection and/or CRISP/ Cas9 experiments. These experiments may involve the generation of pRAP-1 mutated parasites containing deletions and /or substitutions of amino acids in the triad, among other possible experimental approaches. Overall, this experimental approach may provide important clues on the functional relevance of the triad and could be performed using the more developed *B. bovis* model, where gene knockout methods have been established and routinely used^27,28^.

The overall domain organization of the RAP-1 domain, with a globin-like domain followed by a second domain, in this case a 4-helix bundle, generally resembles that of bacterial globin-coupled sensors^26^. However, significant differences between RAP-1 and globin-coupled receptors are observed. First, as mentioned above, RAP-1 seems to lack hemes or other prosthetic groups. Second, the two subdomains of RAP-1, unlike globin-coupled sensors, are tightly associated, including interactions between highly conserved residues between the two subdomains. Whether this tight association is affected by binding of the globin subdomain to protein or small molecule ligands, triggering a response through the closely associated 4-helix bundle subdomain is not known. The high-quality models described here will allow further definition of the function of RAP-1 and its globin-like subdomain.

The second surprising finding was the identification of RAP-1 structural homologues in *Plasmodium* and *Toxoplasma* and other members of the *Sarcocystidae* that share very limited sequence similarity with piroplasmid RAP-1, similarity that is only evident with the assistance of structural alignments. The advent of high-quality structural models for a large number of proteins generated by AF2 and other neural network structural modeling tools will allow extending the identification of commonalities in the proteins involved in parasite infection beyond sequence-based methods. Strikingly, in the case of RAP-1, the structural conservation extends to very specific details including the β-hairpin loop replacing ɑ-helix F. In fact, the unique structural features of piroplasmid RAP-1, especially its globin-like domain, is one of the factors that allowed the identification of distantly related protein family members in other apicomplexan groups. In contrast, the 4-helix bundle region is a more generic protein fold found in many unrelated proteins that would provide less specificity in the search for structural homologues. Other examples of proteins involved in host-cell invasion that would be less amenable to this strategy are the family of kinase-like rhoptry proteins of *Toxoplasma*^29^ that may be hard to distinguish from non-rhoptry kinases and rhoptry proteins with low-quality, unfolded structural models.

In summary, structural analysis using AF2 revealed the presence of globin domains among the members of the pRAP-1 family, and structure-database similarity searches demonstrated that previously unnoticed structural homologous of the members of thepRAP-1 superfamily are present among other related apicomplexan parasites that are important human and animal pathogens. The identification of widely conserved elements in these parasites will enhance our knowledge of the biology of apicomplexan parasites and facilitate the design of methods for controlling diseases that affect humans and animals globally.

## Methods

### RAP-1 structural modeling

The sequences of *B. bovis* RAP-1 (BBOV_IV009860), the globin-coupled sensor of *Geobacter sulfurreducens* (PDB accession 2W31 and WP_010943923.1), *Plasmodium* pRAP-1-structural homologue (PF3D7_1244500) , *C. suis* pRAP-1 structural homologue (CSUI_010388), *N. caninum* (NCLIV_056300), *T. gondii* (TGPRC2312950), *T. annulata* RAP1 R1 RAP1 R2 and R3 ( TA05870), *T. equi* RAP-1 R1, R2, R3 (BEWA_037600, and BEWA_037610), *T. parva* RAP1 R1 *T. parva* RAP1 R2 *T. parva* RAP-1 R3 ( EAN33942, TP01_0704) , *B. bigemina* RAP-1c (AAN84519), *B. bigemina* RAP-1b (AAB72094), *B. divergens* RAP-1 (AEP32236), *B. bigemina* RAP-1 (1906304A), *B. divergens* RRA (AEP32244), *B. divergens* RAP-1b (Bdiv_022630), *B. bovis* RRA (BBOV_IV010280), *B. ovata* RRA (BOVATA_018220), and *B. bigemina* RRA (BBBOND_0107940) were modeled with ColabFold at https://colab.research.google.com/github/sokrypton/ColabFold/blob/main/AlphaFold2.ipynb^18^. Modeling was run using default options for monomeric models without templates and amber relaxation. Models were visualized using PyMOL version 2.5.1 (Schrödinger, LLC). Secondary structure and disulfides were automatically determined by PyMOL.

### Structural alignments

Structural similarities with other structures in the Protein Data Bank or in the AlphaFold Database or between models were determined with the Dali (http://ekhidna2.biocenter.helsinki.fi) and Foldseek servers (https://search.foldseek.com/search)^21,25^.

### Supplementary Information

Supplementary Information 1.

Supplementary Figure 1.

Supplementary Figure 2.

## Data Availability

All data is described in the manuscript.
