# Supplementary material for: Structural definition of babesial RAP-1 proteins identifies a novel protein superfamily across Apicomplexa
Source: Sci Rep. 2023 Dec 15;13:22330. doi: 10.1038/s41598-023-49532-0 (PMC10724250; doi:10.1038/s41598-023-49532-0)
Supplement: Supplementary file 1 — Supplementary Information 1. [file 41598_2023_49532_MOESM1_ESM.docx]

**Supplementary Figure Legends**

**Supplementary Figure 1** - Quality scores of RAP-1 structural models. The pLDDT for the 5 top models are shown along the sequence of each RAP-1 ortholog. The species and ortholog are indicated within each panel.

**Supplementary Figure 2** - RAP-1 model structural alignment scores. DALI Z-scores are shown for each RAP-1 ortholog alignment, shaded in tones of blue according to Z-score. The repeat regions of *Theileria* RAP-1 are aligned individually with the suffix R1-R3 to denote repeat number. *T. equi* RAP-1 refers to BEWA_037600 (XP_004833176) and *T. equi* RAP-1 orf corresponds to BEWA_037610 (XP_004833177). P. falciparum core is the core domain model of Q8I4Y6, without the first 518 amino acids, as shown in Figure 2.
