# Supplementary figures and images for: Structural definition of babesial RAP-1 proteins identifies a novel protein superfamily across Apicomplexa
Source: Sci Rep. 2023 Dec 15;13:22330. doi: 10.1038/s41598-023-49532-0 (PMC10724250; doi:10.1038/s41598-023-49532-0)

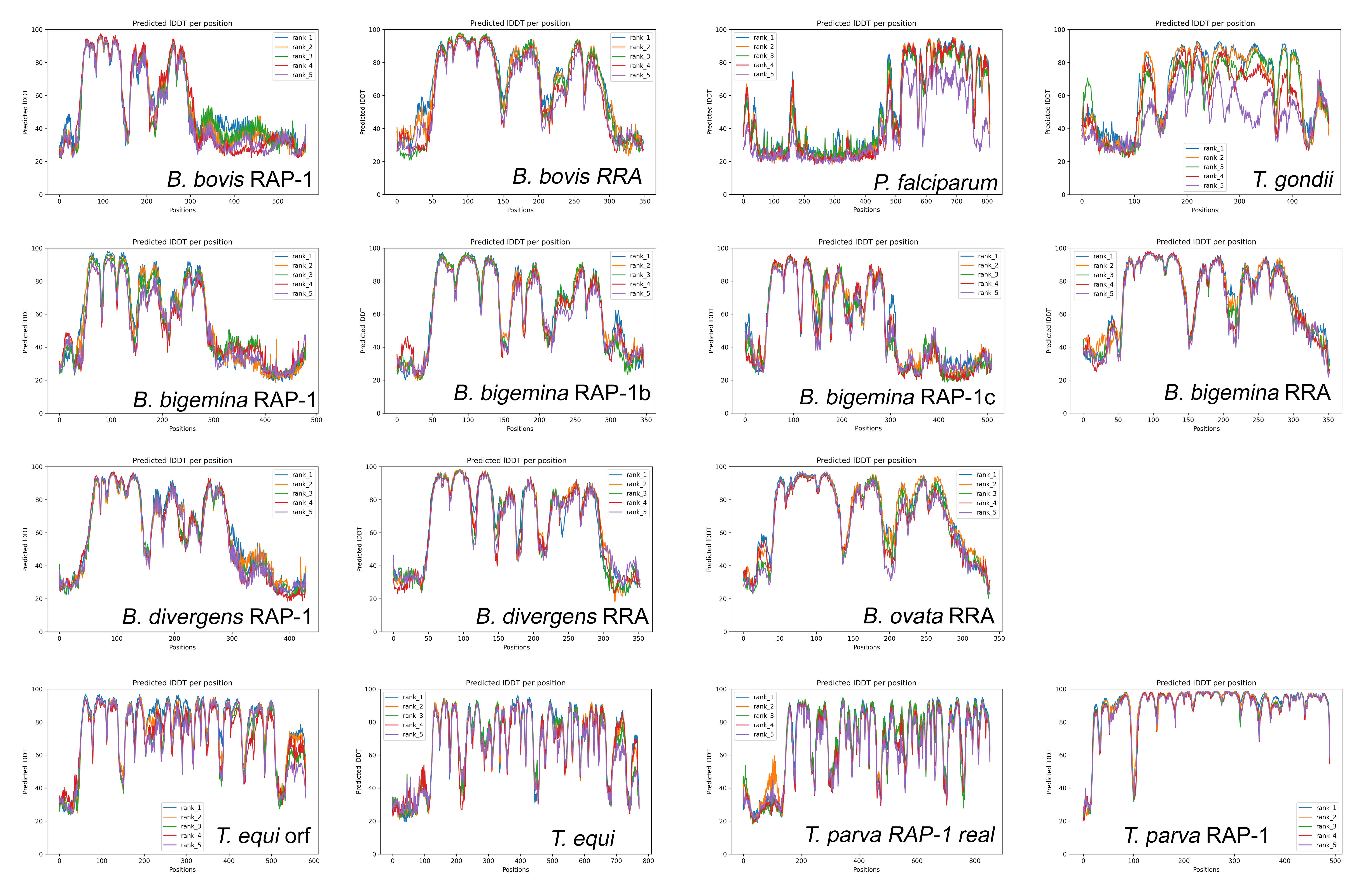

Supplement: Supplementary file 2 — Supplementary Figure 1. [file 41598_2023_49532_MOESM2_ESM.tif]
