# Supplementary material for: Structural definition of babesial RAP-1 proteins identifies a novel protein superfamily across Apicomplexa
Source: Sci Rep. 2023 Dec 15;13:22330. doi: 10.1038/s41598-023-49532-0 (PMC10724250; doi:10.1038/s41598-023-49532-0)
Supplement: Supplementary file 3 — Supplementary Figure 2. [file 41598_2023_49532_MOESM3_ESM.pdf]

	C. suis RAP-1-like	*N. caninum* RAP-1-like	*T. gondii* RAP-1-like	*T. equi* RAP-1 R3	*T. equi* RAP-1orf R2	*T. parva* RAP1 R3	*T. annulata* RAP1 R3	*T. parva* RAP1 R1	*T. annulata* RAP1 R1	*T. equi* RAP-1 R1	*T. parva* RAP1 R2	*T. annulata* RAP1 R2	*T. equi* RAP-1orf R1	*T. equi* RAP-1 R2	*B. bigemina* RAP-1c	*B. bigemina* RAP-1b	*B. divergens* RAP-1	*B. bovis* RAP-1	*B. bigemina* RAP-1	*B. divergens* RRA	*B. divergens* RAP-1b	*B. bovis* RRA	*B. ovata* RRA	*B. bigemina* RRA	*P. falciparum* core
C. suis RAP-1-like		22.1	18.9	5.1	6.7	7.8	7.7	9.8	9.7	9.2	10.2	10.4	10.0	9.8	8.7	7.5	9.4	9.1	9.0	8.1	9.5	8.9	7.3	7.9	6.1
N. caninum RAP-1-like	22.1		25.4	6.1	9.1	10.8	10.5	12.5	12.4	12.3	13.0	13.3	12.5	12.8	11.7	10.2	12.1	11.5	11.0	10.8	11.7	11.9	11.7	11.4	8.7
T. gondii RAP-1-like	18.9	25.4		7.0	8.9	10.2	10.0	11.8	12.2	12.0	12.1	12.6	12.4	12.2	10.7	10.0	10.2	10.4	10.1	10.6	10.4	10.7	10.6	10.9	9.5
T. equi RAP-1 R3	5.1	6.1	7.0		14.4	17.7	19.2	15.2	15.9	16.1	17.6	16.9	16.2	17.9	13.4	14.7	14.4	15.1	14.9	14.0	14.8	15.9	15.4	15.7	9.2
T. equi RAP-1orf R2	6.7	9.1	8.9	14.4		21.6	21.4	19.7	20.3	21.6	22.2	22.2	24.1	22.5	18.4	19.4	18.6	17.1	19.0	17.5	18.8	19.1	18.3	18.3	10.9
T. parva RAP1 R3	7.8	10.8	10.2	17.7	21.6		37.3	20.2	20.9	22.9	23.6	23.7	23.5	24.8	19.7	21.5	21.0	20.1	21.5	20.2	20.8	21.7	20.2	20.4	12.0
T. annulata RAP1 R3	7.7	10.5	10.0	19.2	21.4	37.3		20.0	20.6	22.7	23.8	23.7	23.2	24.9	19.5	21.5	21.0	20.4	21.5	20.3	20.4	21.8	20.5	20.6	12.2
T. parva RAP1 R1	9.8	12.5	11.8	15.2	19.7	20.2	20.0		36.4	25.5	24.4	24.5	25.0	25.1	19.3	20.8	19.1	18.5	20.3	19.3	21.8	21.1	21.1	22.4	11.8
T. annulata RAP1 R1	9.7	12.4	12.2	15.9	20.3	20.9	20.6	36.4		25.9	25.0	24.9	25.3	25.7	19.8	21.3	19.5	19.0	20.6	19.7	21.3	21.1	21.0	21.9	12.2
T. equi RAP-1 R1	9.2	12.3	12.0	16.1	21.6	22.9	22.7	25.5	25.9		26.3	26.0	26.4	26.9	21.3	22.1	22.0	19.8	22.6	22.4	22.9	24.7	24.9	24.6	12.4
T. parva RAP1 R2	10.2	13.0	12.1	17.6	22.2	23.6	23.8	24.4	25.0	26.3		36.0	28.1	29.6	21.4	21.0	20.8	20.6	22.4	20.1	22.0	22.4	22.8	23.1	11.5
T. annulata RAP1 R2	10.4	13.3	12.6	16.9	22.2	23.7	23.7	24.5	24.9	26.0	36.0		28.2	29.4	22.4	21.8	21.4	21.6	22.4	20.3	22.9	22.2	22.4	23.0	11.8
T. equi RAP-1orf R1	10.0	12.5	12.4	16.2	24.1	23.5	23.2	25.0	25.3	26.4	28.1	28.2		29.5	23.2	23.6	22.2	23.5	25.3	21.2	24.8	23.1	23.1	23.2	11.9
T. equi RAP-1 R2	9.8	12.8	12.2	17.9	22.5	24.8	24.9	25.1	25.7	26.9	29.6	29.4	29.5		21.7	22.3	22.8	21.1	23.2	21.0	23.3	24.0	23.7	24.0	11.9
B. bigemina RAP-1c	8.7	11.7	10.7	13.4	18.4	19.7	19.5	19.3	19.8	21.3	21.4	22.4	23.2	21.7		24.7	25.9	26.2	28.6	23.7	27.0	22.8	23.1	23.4	12.7
B. bigemina RAP-1b	7.5	10.2	10.0	14.7	19.4	21.5	21.5	20.8	21.3	22.1	21.0	21.8	23.6	22.3	24.7		24.5	22.9	25.0	22.5	28.1	23.4	22.5	22.9	12.4
B. divergens RAP-1	9.4	12.1	10.2	14.4	18.6	21.0	21.0	19.1	19.5	22.0	20.8	21.4	22.2	22.8	25.9	24.5		27.8	29.3	24.8	27.6	26.4	26.0	25.7	11.9
B. bovis RAP-1	9.1	11.5	10.4	15.1	17.1	20.1	20.4	18.5	19.0	19.8	20.6	21.6	23.5	21.1	26.2	22.9	27.8		34.9	23.6	27.3	23.5	23.7	26.9	10.9
B. bigemina RAP-1	9.0	11.0	10.1	14.9	19.0	21.5	21.5	20.3	20.6	22.6	22.4	22.4	25.3	23.2	28.6	25.0	29.3	34.9		24.9	30.1	25.9	26.2	28.5	11.4
B. divergens RRA	8.1	10.8	10.6	14.0	17.5	20.2	20.3	19.3	19.7	22.4	20.1	20.3	21.2	21.0	23.7	22.5	24.8	23.6	24.9		23.7	28.7	27.8	27.9	12.0
B. divergens RAP-1b	9.5	11.7	10.4	14.8	18.8	20.8	20.4	21.8	21.3	22.9	22.0	22.9	24.8	23.3	27.0	28.1	27.6	27.3	30.1	23.7		26.3	26.6	29.1	11.8
B. bovis RRA	8.9	11.9	10.7	15.9	19.1	21.7	21.8	21.1	21.1	24.7	22.4	22.2	23.1	24.0	22.8	23.4	26.4	23.5	25.9	28.7	26.3		33.7	33.5	12.2
B. ovata RRA	7.3	11.7	10.6	15.4	18.3	20.2	20.5	21.1	21.0	24.9	22.8	22.4	23.1	23.7	23.1	22.5	26.0	23.7	26.2	27.8	26.6	33.7		40.8	11.8
B. bigemina RRA	7.9	11.4	10.9	15.7	18.3	20.4	20.6	22.4	21.9	24.6	23.1	23.0	23.2	24.0	23.4	22.9	25.7	26.9	28.5	27.9	29.1	33.5	40.8		12.0
P. falciparum core	6.1	8.7	9.5	9.2	10.9	12.0	12.2	11.8	12.2	12.4	11.5	11.8	11.9	11.9	12.7	12.4	11.9	10.9	11.4	12.0	11.8	12.2	11.8	12.0	
